# Protective Effects of Wogonin against Alzheimer's Disease by Inhibition of Amyloidogenic Pathway

**DOI:** 10.1155/2017/3545169

**Published:** 2017-06-07

**Authors:** Ding-Siang Huang, Yu-Chen Yu, Chung-Hsin Wu, Jung-Yaw Lin

**Affiliations:** Department of Life Science, National Taiwan Normal University, Taipei, Taiwan

## Abstract

One of the pathogenic systems of Alzheimer's disease (AD) is the formation of *β*-amyloid plaques in the brains of patients, and amyloidogenic activity becomes one of the therapeutic targets. Here, we report wogonin, one of the major active constituting components in* Scutellaria baicalensis*, which has the neuroprotective effects on amyloid-*β* peptides- (A*β*-) induced toxicity. Oral wogonin treatment improved the performance of triple transgenic AD mice (h-APPswe, h-Tau P301L, and h-PS1 M146V) on the Morris water maze, Y-maze, and novel object recognition. Furthermore, wogonin activated the neurite outgrowth of AD cells by increasing neurite length and complexity of Tet-On A*β*_42_-GFP SH-SY5Y neuroblastoma cells (AD cells) and attenuated amyloidogenic pathway by decreasing the levels of *β*-secretase, APP *β*-C-terminal fragment, A*β*-aggregation, and phosphorylated Tau. Wogonin also increased mitochondrial membrane potential (∆*ψ*m) and protected against apoptosis by reducing the expression of Bax and cleaved PARP. Collectively, these results conclude that wogonin may be a promising multifunctional drug candidate for AD.

## 1. Introduction

Alzheimer's disease (AD) is a progressive neurodegenerative disease that is characterized by the aggregation of extracellular amyloid *β* (A*β*) peptides and intracellular neurofibrillary tangles of hyperphosphorylated Tau protein and is resulting in cognitive deficits and memory loss [1, 2]. A*β* plaque is composed of A*β*_42_ and A*β*_40_ peptides that are the major forms in the brain of AD patient. In the amyloidogenic pathway, A*β* generation is mediated by amyloid precursor protein processing that is subsequently proteolysis by *β*-secretase and *γ*-secretase. The *β*-secretase, as *β*-site amyloid precursor protein cleaving enzyme 1 (BACE1), is a rate-limiting enzyme that modulates A*β* production [3, 4], and overproduction of A*β* peptides is known to self-assemble into dimers and high molecular weight oligomers to form fibrils [5–7] that induce neurotoxicity and contribute to AD symptoms [8–10]. Accordingly, inhibition of BACE1 activity is a potent strategy for treating AD.

Mitochondria are targets of A*β* that accumulates in the membrane of mitochondria, and mitochondrial function is disrupted in an early phase of AD [11–15], suggesting that A*β* aggregation is involved in mitochondria-mediated apoptosis [16, 17]. It has been reported that A*β* leads to cytochrome C release through mitochondrial membrane permeabilization [18, 19] that induces mitochondrial-mediated apoptosis.

Wogonin is an active compound of* Scutellaria baicalensis Georgi*, and the effects of wogonin on anti-inflammatory and antioxidation have been studied in various cell types [20–22]. In the present study, the effects of wogonin on BACE1 activity and A*β* oligomerization were investigated. Our findings demonstrated that wogonin inhibited amyloid precursor protein (APP) processing in the *β*-secretase pathway, the phosphorylation of Tau protein, and A*β* aggregation in human neuroblastoma cells and exhibited protective effects on learning and memory impairments in the Morris water maze test, Y-maze, and the novel object recognition test of 3xTg-AD mice.

## 2. Materials and Methods

### 2.1. Materials

A*β*_42_ peptide was obtained from Anaspec (Fremont, CA, USA). 1,1,1,3,3,3-Hexafluoro-2-propanol (HFIP) was purchased from Matrix Scientific (Columbia, SC, USA). Thioflavin T (ThT) was obtained from Abcam (Cambridge, MA, USA). Tris(2,2′-bipyridine)dichlororuthenium(II) hexahydrate (Ru(Bpy)_3_) was purchased from Santa Cruz Biotechnology, Inc. (Dallas, TX, USA). 3-(4,5-Dimethylthiazol-2-yl)-2,5-diphenyltetrazolium bromide (MTT), ammonium persulfate (APS), and retinoic acid (RA) were purchased from Sigma-Aldrich Co. LLC (St. Louis, MO, USA). Dulbecco's Modified Eagle's Medium that contained the F-12 nutrient mixture (DMEM/F12), penicillin/streptomycin,* hygromycin* B, blasticidin S, and The MitoProbe™ JC-1 Assay Kit were obtained from Invitrogen (Grand Island, NY, USA).

Oligomer A11 antibody was obtained from Invitrogen (Grand Island, NY, USA). The antibodies against Bax and cleaved poly (ADP-ribose) polymerase (PARP) were purchased from Cell Signaling Technology, Inc. (Danvers, MA, USA), and those against BACE1, phospho-Tau (Ser396), total Tau (A-10), and actin were obtained from Santa Cruz Biotechnology, Inc. (Dallas, TX, USA). The antibody against *β*-amyloid_1-16_ (6E10) was obtained from Covance Inc. (Princeton, NJ, USA).

### 2.2. Cell Culture

Tet-On A*β*_42_-GFP SH-SY5Y neuroblastoma cells were cultured with DMEM/F12 containing 10% fetal bovine serum, 1% penicillin/streptomycin, 0.1% hygromycin B, and 0.05% blasticidin S at 37°C in a humid 5% CO_2_ environment.

### 2.3. Cell Viability

Cell viability was evaluated by MTT assay by using Tet-On A*β*_42_-GFP SH-SY5Y neuroblastoma cells. Cells were plated in a final volume of 100 *μ*L of medium with 10 *μ*M retinoic acid (RA) in 96-well plates (1 × 10^4^ cells/well). After 24-hour incubation, the cells were treated with various concentrations of wogonin for five days. After the treatment, cells were incubated with 0.5 mg/ml MTT for three hours. The formazan crystals were dissolved by acid-SDS solution (10% sodium* dodecyl sulfate* and 0.01 N HCl), and the absorbance was recorded at a wavelength of 570 nm with an ELISA reader (uQuant, BioTek Instruments, Inc., Winooski, VT, USA).

### 2.4. Acetylcholinesterase (AChE) Activity Assay

Tet-On A*β*_42_-GFP SH-SY5Y neuroblastoma cells were plated in the 6-well plate at density of 1 × 10^5^ cells/well with 10 *μ*M RA and pretreated with wogonin (10 and 25 *μ*M) for 24 hours. Cells were then induced to express A*β*_42_-GFP for five days by treating with 10 *μ*g/mL doxycycline. Briefly, cells were homogenized in ice-cold phosphate-buffered saline (PBS, pH 7.4) and then centrifuged at 13,000 rpm for 20 min at 4°C. The supernatant was performed by Acetylcholinesterase Assay Kit (KA1607, Abnova Corp., Taiwan) where reactions were monitored by measuring the absorbance at 412 nm.

### 2.5. Thioflavin T Assay

The freshly prepared A*β*_42_ peptides (20 *μ*M) with/without wogonin (10 *μ*M) were added in a 100 *μ*L sample containing 50 mM glycine and 40 *μ*M Thioflavin T. The samples were loaded in a 96-well plate and florescent intensity of each sample was measured by excitation of 440 nm and emission of 490 mm every 30 min at 37°C for 16 hours.

### 2.6. Photo-Induced Cross-Linking of Unmodified Proteins (PICUP)

The PICUP was performed as described with a small modification [23]. Briefly, HFIP-treated A*β*_42_ peptide film was dissolved at a concentration of 30 *μ*M in 10% (vol/vol) 60 mM NaOH and 90% (vol/vol) 10 mM sodium phosphate buffer (pH 7.4). The peptide solution was centrifuged at 16,000 ×g for 10 min after 5 min sonication by water bath sonicator. The supernatant was used immediately. 17 *μ*L peptide solution was mixed with 1 *μ*L Ru(Bpy)_3_ (1 mM) and 1 *μ*L APS (20 mM), and the mixture was irradiated for 1 sec with visible light in a closed chamber. The reaction was quenched immediately by adding 1 *μ*L DTT and sample buffer and then kept on ice before analysis by SDS-PAGE.

### 2.7. Dot-Blot Assay

A*β*_42_ was dissolved at a concentration of 2 mM in 100 mM NaOH and followed by water bath sonication for 30 sec, and the A*β*_42_ stock solution was then diluted to 45 *μ*M by adding PBS (pH 7.4) containing 0.02% sodium azide. The A*β*_42_ working solution was incubated with/without 10 *μ*M wogonin at room temperature for different periods (0, 1, and 3 days), and the sample was spotted onto nitrocellulose membrane and filtered on a Bio-Dot apparatus (Bio-Rad). The membrane was blocked by 5% nonfat milk in TBST for 1 hour at room temperature, and then membrane was incubated with A11 polyclonal antibody (1 : 1000) or 6E10 monoclonal antibody (1 : 1000) for overnight at 4°C, followed by incubation with secondary anti-rabbit or anti-mouse antibody conjugated with horseradish peroxidase (1 : 5000) followed by ECL detection.

### 2.8. Western Blot Analysis

After treatments, Tet-On A*β*_42_-GFP SH-SY5Y neuroblastoma cells and cerebral tissue of mice were lysed with radioimmunoprecipitation assay buffer on ice for 10 min, which was followed by sonication and centrifugation at 13,000 rpm at 4°C for 20 min. Equal amounts of the protein samples (40 *μ*g) and the standard protein molecular weight markers were separated by sodium dodecyl sulfate-polyacrylamide gel electrophoresis and blocked with dried 5% skim milk. An immunoblot analysis was performed followed by enhanced chemiluminescence detection.

### 2.9. Mitochondrial Membrane Potential Analysis

Tet-On A*β*_42_-GFP SH-SY5Y cells were plated in 6-well plates at a density of 1 × 10^5^ cells/well with 10 *μ*M RA. After 24 hours, cells were pretreated with 10 *μ*M wogonin for 24 hours and then treated with 10 *μ*g/ml Dox to express A*β*_42_-GFP for five days. Cells were harvested and stained by using JC-1 dye for one hour; then the cells were measured with a flow cytometry (FACSCalibur, Becton Dickinson, San Jose, CA, USA) and analyzed with CellQuest software. A total number of 10,000 cells were recorded for each group.

### 2.10. Quantitative Analysis of Neurite Outgrowth

Neurite outgrowth of Tet-On A*β*_42_-GFP SH-SY5Y cells was measured with a Sholl analysis [24]. The cells were plated in 6-well plates at a density of 5 × 10^4^ cells/well with 10 *μ*M RA, pretreated with 10 *μ*M wogonin for 24 hours, and then induced with 10 *μ*g/mL Dox for five days. The cells were washed with PBS and fixed with 4% paraformaldehyde for 15 min, which were followed by staining with 0.25% (w/v) crystal violet in 2% ethanol/water for 30 min at room temperature. The samples were observed with a microscope, and neurite outgrowth was analyzed as the length of the neurite, while neurite complexity was determined by the number of intersections of the neurites and concentric circles.

### 2.11. Immunohistochemistry (IHC)

3xTG-AD mice were cardiac perfused with phosphate-buffered saline (PBS) and then fixed with 4% formaldehyde (EM grade) at the age of 24 weeks. The brain specimens were embedded in paraffin. Sagittal sections (5 *μ*m) of cortex and hippocampus were then stained with 6E10 antibody (Santa Cruz, California, USA) for 1 hour at room temperature, followed by incubation with biotinylated secondary antibody (Novolink™ Polymer Detection System l; Leica, Wetzlar, Germany) for 30 min at room temperature. The sections were then incubated with avidin-biotin HRP complex (Novolink Polymer Detection System l) for 30 min at room temperature. Finally, visualization was performed with DAB Chromogen (Novolink Polymer Detection System l) and counterstained with hematoxylin (Novolink Polymer Detection System l) following supplier's protocol.

### 2.12. Animal Model

C57BL/6 Non-Tg wild-type mice and homozygous 3xTg-AD transgenic mice harboring human PS1_M146V_, human APP_Swe_, and human tan_P301L_ [25] were bred and maintained in the animal facility at National Taiwan Normal University (NTNU) under specific pathogen-free conditions in accordance with the Institutional Guidelines of the Animal Care and Use Committee at NTNU. Three groups of mice were used and injected [intraperitoneally (*i.p.*)] with the vehicle or test compounds every other day from the 8th week to the 24th week of age. The first group (8 wild-type mice) and second group (8 3xTg-AD mice) were injected (*i.p.*) with dimethyl sulfoxide (DMSO), and the third group (8 3xTg-AD mice) was injected (*i.p.*) with wogonin (10 mg/kg).

The animals were examined by using behavioral tasks: (a) the Morris water maze test when the animals were 16- and 24-week-old, (b) spontaneous alternation behavior Y-test when they were 24 weeks old, and (c) the novel object recognition task test when they were 23 weeks old. After completion of the behavioral tasks, the mice were sacrificed, and their brain tissues were homogenized with PBS and protease inhibitors for western blot and immunohistochemistry analyses. The body weights of the animals were measured every two weeks from the 6th to 24th week of age.

### 2.13. Data Analysis

All data are expressed as mean ± SEM, and the statistical analyses were carried out using one-way ANOVA followed by Tukey's post hoc tests. Differences were considered statistically significant at *p* < 0.05.

## 3. Results

### 3.1. Wogonin Suppressed the A*β*_42_-Induced Amyloidogenic Pathway

**Figure 1:**
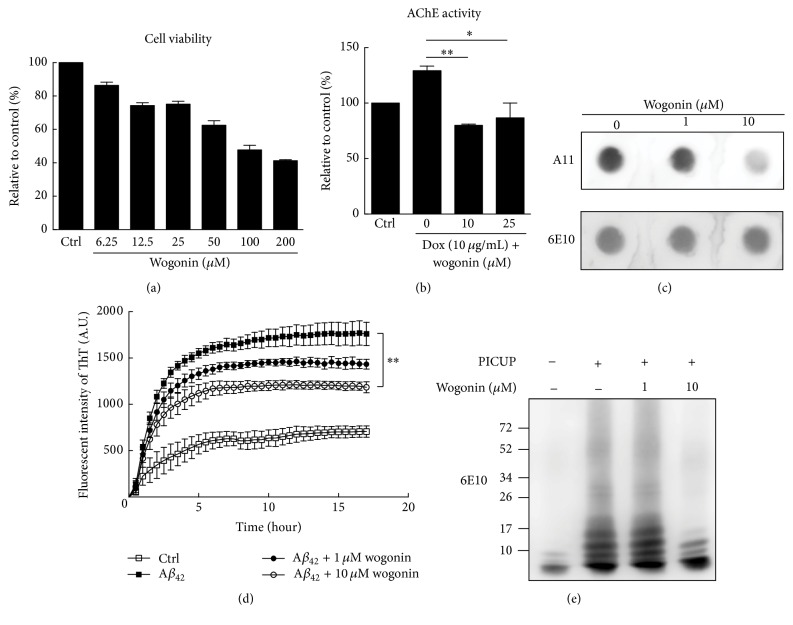
Effects of wogonin on AChE activity, A*β*_42_ oligomerization, and fibrillization. (a) Tet-On A*β*42-GFP SH-SY5Y cells were treated with various concentrations of wogonin (6.25–200 *μ*M), and cell viability was measured by MTT assay. (b) Tet-On A*β*_42_-GFP SH-SY5Y cells were induced with 10 *μ*g/mL doxycycline in the absence or presence of wogonin (10 and 25 *μ*M) for five days, and AChE activity was then measured by Acetylcholinesterase Assay Kit. (c) A*β*_42_ (45 *μ*M) was incubated in the absence or presence of wogonin (1 and 10 *μ*M) at room temperature for 3 days and then performed by dot-blot assay. A*β*_42_ oligomers were detected by using oligomer A11 polyclonal antibody, and total Α*β*_42_ were performed by using 6E10 antibody. (d) HFIP-treated A*β*_42_ (20 *μ*M) was incubated in the absence or presence of wogonin (1 and 10 *μ*M) assayed by Thioflavin T (ThT), and the fluorescent intensity (excitation/emission = 440 nm/490 nm) was recorded every 30 min for 16 hours at 37°C. (e) A*β*_42_ (25 *μ*M) in the absence or presence of wogonin (1 *μ*M or 10 *μ*M) was subjected to photo-induced cross-linking unfolded proteins (PICUP) and SDS-PAGE analysis to examine A*β* oligomerization. ^*∗*^*p* < 0.05, ^*∗∗*^*p* < 0.01.

To examine the half maximal inhibitory concentration (IC_50_) of wogonin, Tet-On A*β*_42_-GFP SH-SY5Y neuroblastoma cells were cultured in the absence and presence of wogonin at various concentrations (6.25–200 *μ*M). As shown in Figure 1(a), the IC_50_ of wogonin was about 100 *μ*M determined by MTT assay. The deficit in cholinergic system has been found in AD patients and associated with memory deficits [26]. Subsequently, acetylcholinesterase (AChE) inhibitors were used for symptomatic treatment of AD [27]. In Figure 1(b), AChE activity was significantly inhibited in the presence of wogonin (10 *μ*M). The amyloidogenic pathway results in A*β*_42_ assembly, which is the main source of the toxicity of neurons. Wogonin (10 *μ*M) inhibited A*β*_42_ oligomers species by performing dot-blot assay (Figure 1(c)). Here, we examined A*β*_42_ fibrillization by Thioflavin T (ThT) assay in the absence and presence of wogonin (1 and 10 *μ*M). Data showed that 10 *μ*M wogonin significantly inhibited A*β* fibrillization (Figure 1(d)). To examine whether wogonin disrupts A*β*_42_ assembly in the starting time point, photo-induced cross-linking of unfolded protein (PICUP) was performed in the presence of wogonin. We found that 10 *μ*M wogonin potently disrupted A*β*_42_ assembly (Figure 1(e)). These data indicated that wogonin interfered with A*β*_42_ oligomerization and fibrillization in vitro.

### 3.2. Wogonin Attenuated A*β*_42_-Induced Apoptosis

**Figure 2:**
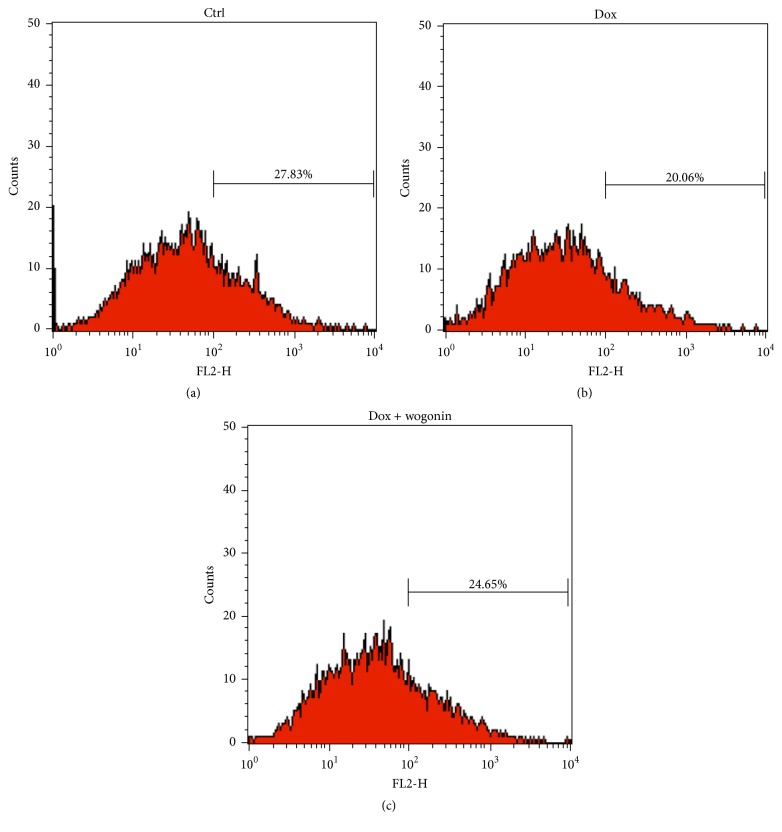
Wogonin rescued the loss of the mitochondrial membrane potential in Tet-On A*β*_42_-GFP SH-SY5Y cells. A*β*_42_-GFP SH-SY5Y cells were incubated with/without 10 *μ*M wogonin for one day at a density of 1 × 10^5^, and then cells were induced with 10 *μ*g/mL doxycycline (Dox) to express A*β*_42_ for five days. The cells were treated with vehicle as a control, and the loss of Δ*ψm* was measured by JC-1 staining with a flow cytometer. The treatments included (a) control, (b) Dox (10 *μ*g/mL), and (c) Dox with wogonin (10 *μ*M).

In order to examine whether wogonin protects Tet-On A*β*_42_-GFP SH-SY5Y cells against mitochondria-mediated apoptosis, the effects of wogonin on the mitochondrial potential (Δ*ψm*) of A*β*_42_-GFP SH-SY5Y cells were examined. The induced cells exhibited a 27.9% decrease in Δ*ψm* compared to that of uninduced cells (Figures 2(a) and 2(b)), and pretreatment with wogonin (10 *μ*M) resulted in 11.4% decrease (Figure 2(c)).

**Figure 3:**
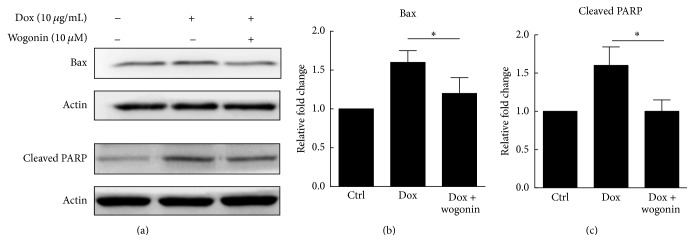
Wogonin decreased the expression levels of Bax and cleaved PARP in Tet-On A*β*_42_-GFP SH-SY5Y cells. (a) A*β*_42_-GFP SH-SY5Y cells were pretreated with/without 10 *μ*M wogonin for one day, and then cells were induced by 10 *μ*g/mL doxycycline (Dox) for five days. After treating with 10 *μ*M wogonin for one days, 3 × 10^5^ A*β*_42_-GFP SH-SY5Y cells were induced with 10 *μ*g/mL Dox for five days to analyze the expression levels of Bax and cleaved poly (ADP-ribose) polymerase (PARP) by western blotting. Quantitative analyses of the levels of Bax (b) and cleaved PARP (c) were conducted with ImageJ respectively, and actin was used as a loading control. The results are shown as mean ± SEM, *n* = 3, ^*∗*^*p* < 0.05.

Furthermore, the expression levels of mitochondrial function biomarkers, such as Bax, were investigated by western blot analysis. In A*β*_42_-GFP-expressing cells, the expression level of Bax was increased to 1.6-fold compared to that of control cells, and it decreased to 1.2-fold after treatment with 10 *μ*M wogonin (Figures 3(a) and 3(b)).

Because wogonin reduced the Δ*ψm*, the effects of wogonin on the activation of cleaved PARP were examined. As shown in Figure 3, the treatment of A*β*_42_-GFP-expressing cells with 10 *μ*g/mL Dox increased the level of cleaved PARP by 1.5-fold, and treatment with wogonin (10 *μ*M) decreased the level of cleaved PARP to 1.0-fold (Figure 3(c)). These findings suggest that wogonin alleviated the apoptosis activation of that which was induced by A*β*_42_ cytotoxicity.

### 3.3. Wogonin Increased Neurite Outgrowth

**Figure 4:**
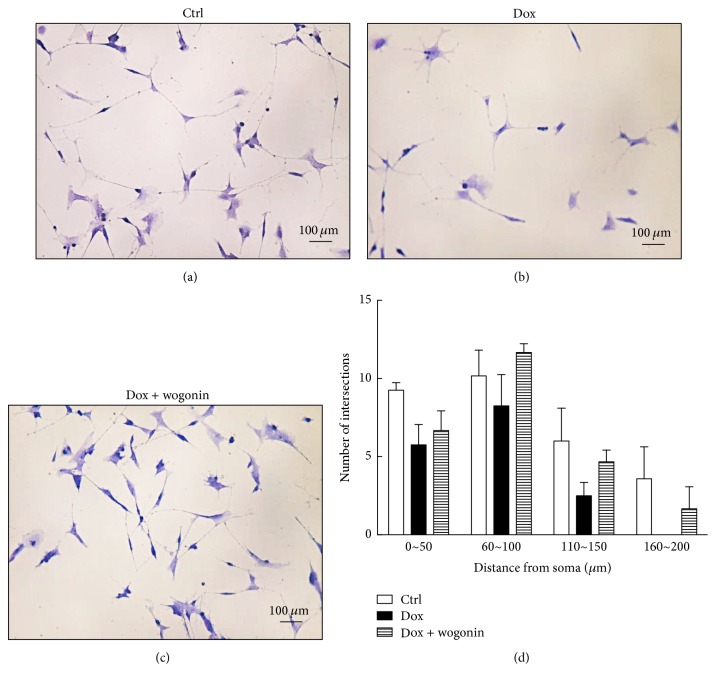
Wogonin improved neurite outgrowth in Tet-On A*β*_42_-GFP SH-SY5Y cells. A*β*_42_-GFP SH-SY5Y cells at a density of 5 × 10^4^ were pretreated with 10 *μ*M wogonin for one day, which was followed by the addition of 10 *μ*g/mL Dox in order to induce A*β*_42_-GFP expression for five days. The cells were treated with vehicle as a control, and cell morphology of control (a), Dox (b), and Dox with wogonin (c) was examined by Sholl analysis to quantify neurite length and complexity. The results are shown as mean ± SEM, *n* = 3. Scale bar: 100 *μ*m.

In the A*β*_42_-GFP SH-SY5Y cells treated with 10 *μ*g/mL Dox to induce A*β*_42_-GFP expression, neurite length was less than 160 *μ*m from the soma (Figures 4(a) and 4(b)), and the number (mean ± standard error) of intersections was 8.3 ± 2.3. After wogonin (10 *μ*M) treatment, the neurite length and the number of intersections increased to 200 *μ*m and 11.7 ± 0.7 (Figures 4(c) and 4(d)), respectively. These findings indicate that wogonin significantly increased the neurite outgrowth and complexity of AD cells.

### 3.4. Wogonin Ameliorated the Memory Deficit in 3xTg-AD Transgenic Mice

**Figure 5:**
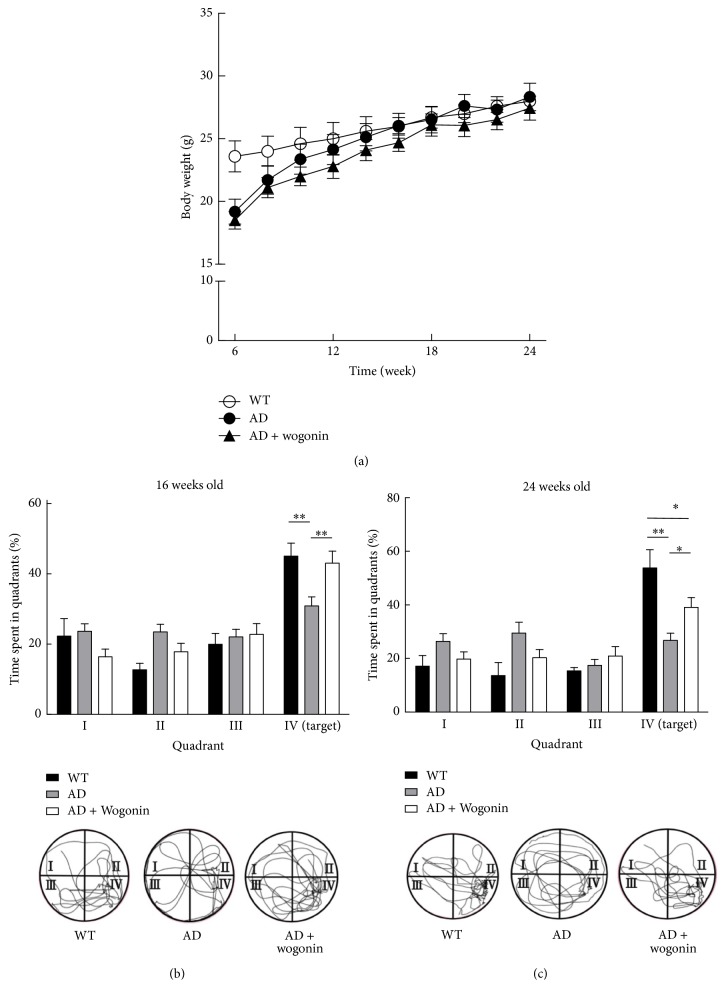
Wogonin alleviated the memory deficits in eight 3xTg-AD mice. Mice were injected [intraperitoneally (*i.p.*)] with 10 mg/kg wogonin every other day from the 8th to the 24th week of age. (a) The body weights of the mice were measured every 2 weeks from the 6th to the 24th week of age. (b) 16-week-old mice were subjected to water maze experiments without platform, and the time spent in quadrants was measured. Representative path tracing was presented in the lower panel. (c) Probe trial of water maze experiments was conducted in the 24-week-old mice, and the time spent in quadrants was measured. Representative path tracing was presented in the lower panel, and the results are shown as mean ± SEM, *n* = 8, ^*∗*^*p* < 0.05, ^*∗∗*^*p* < 0.01.

Based on previous report, the LD_50_ of wogonin was found to be 3.9 g/kg [28], and we used the dose of wogonin at 10 mg/kg, for* i.p.* injection from 8th week to 24th week of age. The body weights of the 3xTg-AD transgenic mice did not differ from those of the control mice significantly (Figure 5(a)). In order to investigate whether wogonin rescues the memory deficit in 3xTg-AD mice, a Morris water maze experiment was carried out in order to evaluate hippocampal-dependent reference memory in the 16- and 24-week-old mice. The platform was removed in order to perform a probe trial by recording the path length and time spent in the quadrants. The results indicated that wogonin-treated AD mice spent significantly more time in the targeted quadrant than the vehicle-treated AD mice at 16th and 24th week (1.4-fold and 1.5-fold, resp.). These findings suggest that wogonin markedly ameliorated the memory impairments of AD mice (Figures 5(b) and 5(c)).

**Figure 6:**
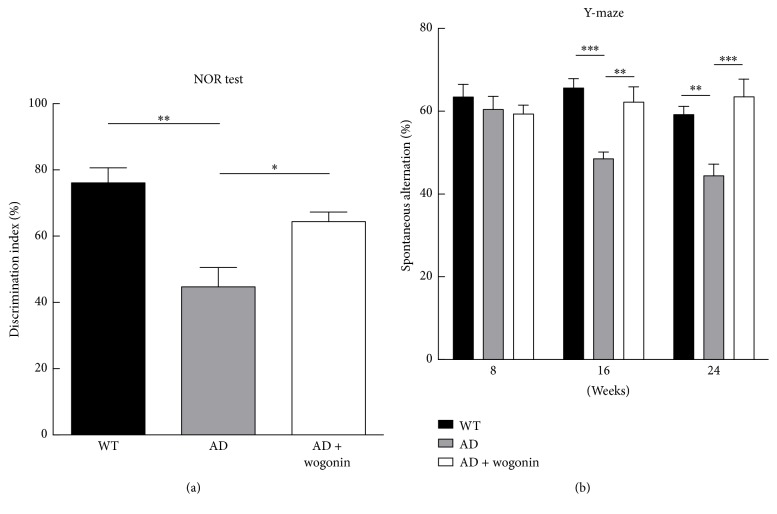
Wogonin treatment attenuated the behavior on the novel object recognition task and Y-maze test in eight 3xTg-AD mice. 3xTg-AD mice were applied 10 mg/kg wogonin every other day from the 8th to the 24th week of age by intraperitoneal injection. (a) Novel object recognition (NOR) experiments were performed in the 23-week-old mice. The discrimination index was calculated as a percentage ratio of* T*_B_/(*T*_A_ +* T*_B_) × 100. (a) Familiar object. (b) Novel object. (b) Spontaneous alternation behavior Y-maze tests were performed in the 8-, 16-, and 24-week-old mice. Alternation (%) = [(number of alternations)/(total arm entries − 2)] × 100. All of the values are shown as mean ± SEM, *n* = 8, ^*∗*^*p* < 0.05, ^*∗∗*^*p* < 0.01, and ^*∗∗∗*^*p* < 0.001.

The novel object recognition test, which involves the frontal cortex, entorhinal cortex, and hippocampus, was employed to examine short-term memory. The results suggested that AD mice treated with vehicle did not preferentially explore the novel object, and the discrimination index was less than 50%. In contrast, wogonin-treated AD mice displayed 62.8% increment in discrimination index compared to that of AD mice for the novel object at the 23rd week (Figure 6(a)). Y-maze test was conducted in order to examine short-term and working memory. The results indicated that vehicle-treated AD mice displayed a decrement of spontaneous alternation behavior, whereas wogonin-treated AD mice showed a 1.3-fold and 1.4-fold increment in spontaneous alternation at the 16th and 24th week, respectively (Figure 6(b)). These results suggest that wogonin significantly recovered the short-term memory of AD mice.

### 3.5. Wogonin Attenuated the Activation of the Amyloidogenic Pathway in the Cerebral Tissues of 3xTg-AD Mice

**Figure 7:**
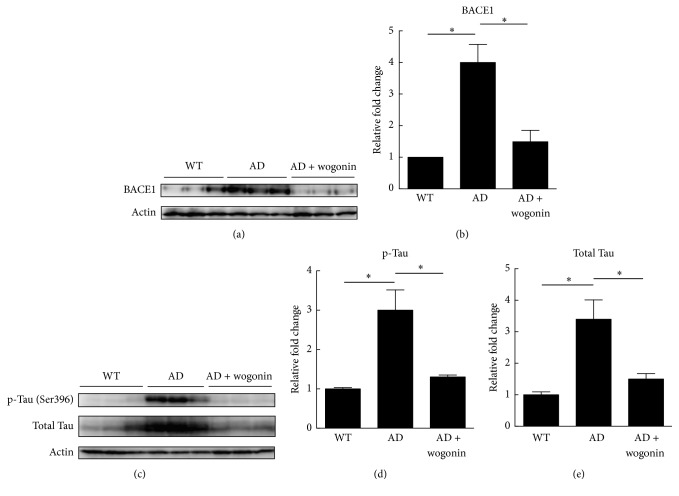
Wogonin decreased the levels of BACE1, p-Tau, and total Tau proteins in the cerebral tissue of three 3xTg-AD mice. Mice were given intraperitoneal injection with vehicle/wogonin (10 mg/kg) every other day from the 8th to the 24th week of age, and mice were sacrificed for western blot analysis in the 24th week of age. (a) The expression level of BACE1 in the cerebra of mice was measured using western blot analysis in the 24-week-old mice. (b) Quantitative analysis of the level of BACE1 was executed with ImageJ. (c) The cerebellar extracts of 3xTg-AD mice were immunostained against p-Tau and Tau protein in the 24-week-old mice. Relative intensity of p-Tau (d) and total Tau (e) was performed with ImageJ. Actin was used as a loading control. The results are shown as mean ± SEM, ^*∗*^*p* < 0.05.

The expression levels of APP and BACE1, which are related to the amyloidogenic pathway, were investigated in the cerebral tissues of AD mice in order to examine whether wogonin treatment inhibits the amyloidogenic pathway. Western blot analyses demonstrated that the expression level of BACE1 in vehicle-treated AD mice was 4.0-fold (Figures 7(a) and 7(b)), higher than that of wild-type mice, but the level in wogonin-treated AD mice was only 1.5-fold.

**Figure 8:**
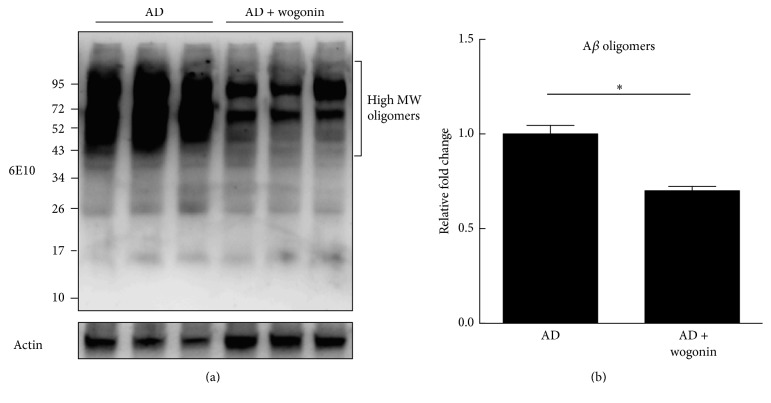
Wogonin decreased A*β* aggregates (high molecular weight oligomers) in the cerebral tissue of three 3xTg-AD mice. 3xTg-AD mice were given injection intraperitoneally with vehicle/wogonin (10 mg/kg) every other day from the 8th to the 24th week of age. Mice were sacrificed when they were 24 weeks old, and cerebellar extracts were subjected to western blot analysis. (a) Aggregation of A*β* on cerebellar extracts of 3xTg-AD mice was measured by using a 6E10 antibody. (b) Relative intensity of A*β* aggregates was presented with ImageJ. Actin was used as a loading control. The results are shown as mean ± SEM, ^*∗*^*p* < 0.05.

In addition, the expression levels of phospho-Tau and total Tau were examined. The levels of phospho-Tau and total Tau in the vehicle-treated AD mice were 3.0- and 3.4-fold, respectively, higher than those of wild-type mice. Consistently, wogonin decreased the expression levels of phospho-Tau and total Tau to 1.3- and 1.5-fold, respectively (Figures 7(c), 7(d), and 7(e)), than those of AD mice. In addition, high molecular weight oligomers (>40 kDa) of wogonin-treated AD mice were decreased to 0.7-fold compared to that of vehicle-treated AD mice (Figures 8(a) and 8(b)). These results strongly suggested that wogonin profoundly suppressed the amyloidogenic pathway, as well as Tau and phosphorylated Tau protein, in the cerebra of AD mice.

**Figure 9:**
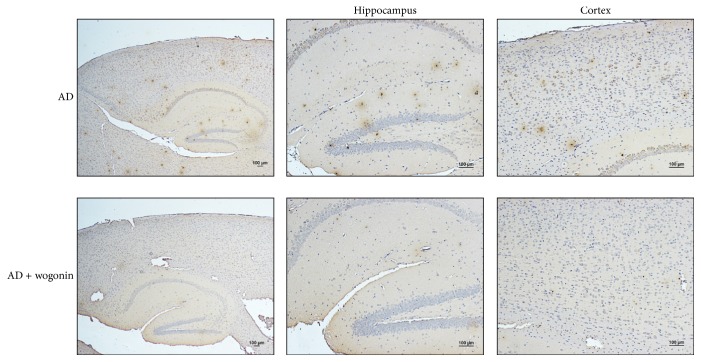
Treatment with wogonin reduces amyloid plaques in the cortex and hippocampus in 3xTg-AD mice. 3xTg-AD mice were applied with vehicle/10 mg/kg wogonin (*i.p.* injection) every other day from the 8th to the 24th week of age. Mice were sacrificed at the 24th week of age, and paraffin-embedded brain sections were subjected to immunohistochemical staining. (a) Immunohistochemical staining of sections from 24-week-old 3xTg-AD mice was stained with A*β* (6E10) antibodies in the cortex and hippocampus. Scale bar: 100 *μ*m.

Because the formation of amyloid plaque is a hallmark of AD, we used immunohistochemical analysis to determine whether wogonin decreases A*β* deposition in the brain of AD mice. A*β* immunoreactive plaques were observed using the 6E10 antibody in the cortex and hippocampus of vehicle-treated AD mice at 24th week of age, and the treatments with wogonin reduced the number and the area of A*β* immunoreactive plaques compared to that of control group (Figure 9). It indicated that wogonin attenuated A*β* aggregation in the cortex and hippocampus of AD mice at 24th week.

## 4. Discussion 

The amyloid cascade hypothesis that A*β* aggregates form amyloid plaque, neurofibrillary tangle, and lead to neuronal death is one of pathogeneses in AD [29]. However, it has shown that soluble oligomers of A*β* peptide are the key factors that contribute to neurotoxicity, synaptic loss, and memory impairment in AD [30–34], and inhibition of A*β* aggregates attenuates the toxicity [35, 36].

Therefore, one of therapeutic strategies for AD is to interfere with A*β* aggregates. In the present study, the oligomeric assembly of A*β*_42_ peptide and the level of A*β* fibril formation are disturbed by wogonin in vitro, suggesting that wogonin inhibits nucleated oligomerization and seeding mediated aggregation. As cholinergic deficit is observed in the early stages of AD and associated with memory deficits, 10 *μ*M wogonin (1/10 IC_50_) significantly inhibited AChE activity in Tet-On A*β*_42_-GFP SH-SY5Y neuroblastoma cells, suggesting that wogonin disrupted the activation of AChE induced by A*β*_42_.

Several animal models have been generated to develop AD-like pathology in order to study the disease modifying effects of potential treatment of AD. In the present study, we examined the effects of wogonin on the cerebral tissues of 3xTg-AD mice, overexpressing human APP_Swe_, human tau_P301L_, and human PS1_M146V_ mutation. This model demonstrates an age-dependent onset of AD with deficits in synaptic plasticity and cognition correlating with the deposition of intracellular A*β* in the early stage, extracellular amyloid plaques at 6 months beginning with the frontal cortex, and then expanded to the hippocampus, as well as other cortical regions, and neurofibrillary tangles observed in moderate to severe stages [25, 37, 38]. Here, we show that wogonin inhibits amyloid plaque burden in the cortex and hippocampus of 3xTg-AD mice. Furthermore, A*β* oligomers are dramatically decreased by wogonin administration in the hippocampus extraction of 3xTg-AD mice.

It has been demonstrated that inhibition of amyloidogenic pathway by decreasing BACE1 alleviates amyloid pathology in mouse model of AD [39, 40]. We show that BACE1 expression is decreased by wogonin in vivo and in vitro model of AD. Taken together, these date suggest that wogonin inhibits the amyloidogenic processing by decreasing BACE1 expression.

Neuronal death underlies the symptoms of several neurodegenerative diseases, including Parkinson's disease and Alzheimer's disease [41]. Several studies indicate that A*β* localizes to mitochondria [11, 15, 42], and the accumulation of intracellular A*β* has been known to result in neuronal apoptosis, which is associated with mitochondrial dysfunction [11, 17, 43–47]. Bcl-2 family proteins are involved in mitochondrial related apoptosis by regulating the mitochondrial membrane permeability. Bax, a proapoptotic protein, is localized to the mitochondrial outer membrane and increases membrane permeability, which is resulting in cell death [48, 49]. In the present study, protein level of Bax is decreased by wogonin in vitro and in vivo model of AD. Furthermore, wogonin inhibits the loss of mitochondrial membrane potential induced by A*β* in Tet-On A*β*_42_-GFP SH-SY5Y cells. Activation of apoptotic pathway in response to mitochondrial dysfunction in Tet-On A*β*_42_-GFP SH-SY5Y cells is decreased in the presence of wogonin by suppressing cleaved caspase-9 and cleaved PARP.

Progressive decline in cognition is most obvious symptom in AD patient. Several behavioral tests are used to evaluate cognitive ability in mice. In this study, wogonin improves the spatial reference memory measured by Morris water maze at the age of 16 weeks and 24 weeks old. However, the spatial reference memory has no difference between WT mice and wogonin-treated mice at the age of 16 weeks old, but it is impaired at the age of 24 weeks. It is indicating that reference memory impairment is delayed by wogonin in 3xTg AD mice. On the other hand, the working memory is measured by Y-maze and novel object recognition test. Wogonin alleviates the working memory impairment in 3xTg AD mice.

It has been demonstrated that Α*β* can induce cognitive impairment and trigger mitochondria-mediated apoptosis by upregulation of Bax [50, 51]. Here we found that wogonin inhibits the expression levels of BACE1, A*β* oligomer, Bax, and cleaved caspase-9, suggesting that wogonin improves cognition through inhibition of A*β* oligomerization and mitochondria-mediated apoptosis in 3xTg AD mice.

Taken together, wogonin has various neuroprotective and neurotrophic activities, including inducing neurite outgrowth. In addition, wogonin alleviates the cognitive deficits of 3xTg AD mice. Therefore, the wogonin-regulated APP processing serves as a neuroprotective activity that might contribute to the treatment of AD.
